# A Deployable and Cost-Effective Kirigami Antenna for Sub-6 GHz MIMO Applications

**DOI:** 10.3390/mi13101735

**Published:** 2022-10-13

**Authors:** Saad Hassan Kiani, Mohamed Marey, Umair Rafique, Syed Imran Hussain Shah, Muhammad Adil Bashir, Hala Mostafa, Sai-Wai Wong, Naser Ojaroudi Parchin

**Affiliations:** 1 Smart Systems Engineering Laboratory, College of Engineering, Prince Sultan University, Riyadh 11586, Saudi Arabia; 2 Department of Electrical Engineering, IIC University of Technology, Phnom Penh 121206, Cambodia; 3 Department of Information Engineering, Electronics and Telecommunications, Sapienza University of Rome, 00184 Rome, Italy; 4 Faculty of Electronics, Telecommunications and Informatics, Gdańsk University of Technology, 80-233 Gdańsk, Poland; 5 Electrical Engineering Department, Bahauddin Zakariya University, Multan 60800, Pakistan; 6 Department of Information Technology, College of Computer and Information Sciences, Princess Nourah bint Abdulrahman University, P.O. Box 84428, Riyadh 11671, Saudi Arabia; 7 College of Electronics and Information Engineering, Shenzhen University, Shenzhen 518060, China; 8 School of Engineering and the Built Environment, Edinburgh Napier University, Edinburgh EH10 5DT, UK

**Keywords:** MIMO, Kirigami antenna, sub-6 GHz, polarization diversity

## Abstract

In this work, a low-cost, deployable, integratable, and easy-to-fabricate multiple-input multiple-output (MIMO) Kirigami antenna is proposed for sub-6 GHz applications. The proposed MIMO antenna is inspired by Kirigami art, which consists of four radiating and parasitic elements. The radiating and parasitic elements are composed of a rectangular stub. These elements are placed in such a way that they can provide polarization diversity. The proposed MIMO antenna is designed and fabricated using a soft printed board material called flexible copper-clad laminate (FCCL). It is observed from the results that the proposed MIMO antenna resonates in the 2.5 GHz frequency band, with a 10 dB reflection coefficient bandwidth of 860 MHz ranging from 2.19 to 3.05 GHz. It is worthwhile to mention that the isolation between adjacent radiating elements is higher than 15 dB. In addition, the peak realized gain of the MIMO antenna is around 11 dBi, and the total efficiency is more than 90% within the band of interest. Moreover, the envelope correlation coefficient (ECC) is noted to be less than 0.003, and the channel capacity is ≥17 bps/Hz. To verify the simulated results, a prototype was fabricated, and excellent agreement between the measured and computed results was observed. By observing the performance attributes of the proposed design, it can be said that there are many applications in which this antenna can be adopted. Because of its low profile, it can be used in 5G small-cell mobile MIMO base stations, autonomous light mobility vehicles, and other applications.

## 1. Introduction

In the last decade, there has been an increasing demand for high-gain and deployable antennas with a wide beamwidth, especially in the defense sector. This is because traditional bulky parabolic antennas typically require heavy vehicular transport by land or air; thus, low-cost and lightweight antennas are required. Antennas based on foldable structures can offer unique performance characteristics in comparison with those of traditional radiating structures [1,2]. Some such examples are Origami- and Kirigami-type structures [3,4,5]. These are low-cost antennas that offer unique performance characteristics in comparison with those of traditional radiating structures. The primary difference between Kirigami and Origami is their folding and unfolding characteristics. Origami technology is based on the folding and unfolding of a structure, whereas Kirigami structures are formed through the introduction of cuts before the folding and unfolding states. As a result, an intended geometry can be realized by introducing positional cuts in a specific manner on a flexible substrate, such as polyethylene terephthalate (PET), fire-protective covering (FPC) sheets, or paper [5,6,7,8,9].

In the literature, only a few Kirigami and Origami antennas have been reported. In [4], a thick and foldable Origami-based patch antenna array was designed for the 2.45 GHz frequency band. The designed array consisted of four patch elements, which were fed differentially. The folded configuration of the designed array was achieved using a surrogate hinge architecture. As the Origami state changed from unfolded to folded, a beam-switching capability was observed up to a scanning angle of 35${}^{\circ }$. Using chemical sintering technology, the authors of [6] demonstrated a dual-band Sirenpinski-shaped flexible antenna. The proposed inkjet antenna was made on a thin paper sheet, generating resonance bandwidths of 1.5–2.7 and 5.1–11 GHz. The antenna radiation characteristics resembled dipole characteristics at lower frequencies, and at higher frequencies, the radiation patterns became slightly directive. Similarly, in [7], an RFID system with an approximately 700 MHz bandwidth was designed on a paper substrate using inkjet printing. To increase the flexibility characteristics, surface modification and electroless deposition were applied. The inkjet technology has its limitations, as the active catalyst ink likely penetrates into flexible substrates, leaving a small amount of dilute ink on the surface. This results in the slow removal of copper from printed patterns.

In [8], a flexible multilayer folded strip monopole antenna exhibiting a 10 dB impedance bandwidth of 2–3 GHz was presented. The antenna was designed on two different flexible PET and glossy paper substrates. A co-planar waveguide array was presented using graphene-printed technology for radar systems [9]. The peak gain value obtained for the proposed array was 4.8 dBi, with bandwidths ranging from 4.7 to 8 GHz. In [10], a pop-up mechanically controlled reconfigurable Kirigami MIMO antenna was presented. The pop-up Kirigami antenna’s resonance response was controlled with an SMA actuator spring. The antenna resonated from 2.41 to 2.5 GHz and from 3.2 to 3.3 GHz. The gain of the antenna varied in the range of 5.9 to 6.4 dBi. In addition, the isolation among MIMO elements was noted to be 10 dB. In [11], a Kirigami antenna was presented with a frequency ranging from 0.47 to 0.57 GHz (−6 dB criteria) with a peak gain of 1.53 dB. In [12], a quasi-Yagi monopole antenna was designed using Origami magic spiral cubes, which could easily be used in the unfolded and folded states. The quasi-Yagi antenna consisted of an L-shaped reflector and a driven monopole, as well as two L-shaped directors. The L-shaped reflector helped in increasing the gain of the antenna from 1.9 to 5.7 dBi, and the addition of other directors further enhanced the gain to 7.3 dBi.

In [13], a frequency-reconfigurable dipole antenna was presented using an Origami flasher. First, the dipole element was designed on flat paper, and then it was folded into a cube-like shape, which was related to the Origami flasher. In the folded state, the length of the dipole element was extended due to the patch pole, but this extension behaved as a dummy in the unfolded state. From the presented results, it was observed that the designed Origami antenna operated at 1.23 GHz in the unfolded state and at 0.77 GHz in the folded state. It was also observed from the results that the gain and efficiency of the antenna decreased in the folded state. In [14], a pattern-reconfigurable Kirigami antenna for the 3.5 GHz frequency band was presented. For enhanced gain, two Kirigami parabolic reflectors were utilized. It was observed that the Kirigami reflector enhanced the gain of an antenna by 2.08 dB compared to the planar reflector. In [15], a two-element dual-band Origami MIMO antenna was presented for WLAN 2.45 and 5.2 GHz applications. The peak gain noted at both resonances was 1.4 and 2.78 dBi, respectively.

Although the presented designs offered good performance attributes, they consisted of a single-input–single-output (SISO) configuration. Therefore, they were unable to handle multipath fading. Due to the advantages posed by Kirigami and Origami structures, it is reasonable to study these technologies for MIMO systems. Therefore, in this paper, a simple Kirigami-based MIMO antenna with a foldable pop-up card is proposed. The proposed MIMO antenna consists of four monopoles, each with a parasitic element. The results show that the designed MIMO antenna resonates well at the 2.5 GHz frequency band, which is one of the major candidate bands for sub-6 GHz 5G applications. Furthermore, the designed MIMO antenna offers a 10 dB impedance bandwidth of 860 MHz and an 11 dBi peak gain. In addition, the efficiency and the ECC of the system are around 90% and 0.003, respectively. Based on the performance characteristics, it is believed that the proposed MIMO antenna could be a potential candidate for space, military, and other microwave applications where deployability is the main concern.

## 2. Kirigami MIMO Antenna

**Figure 1:**
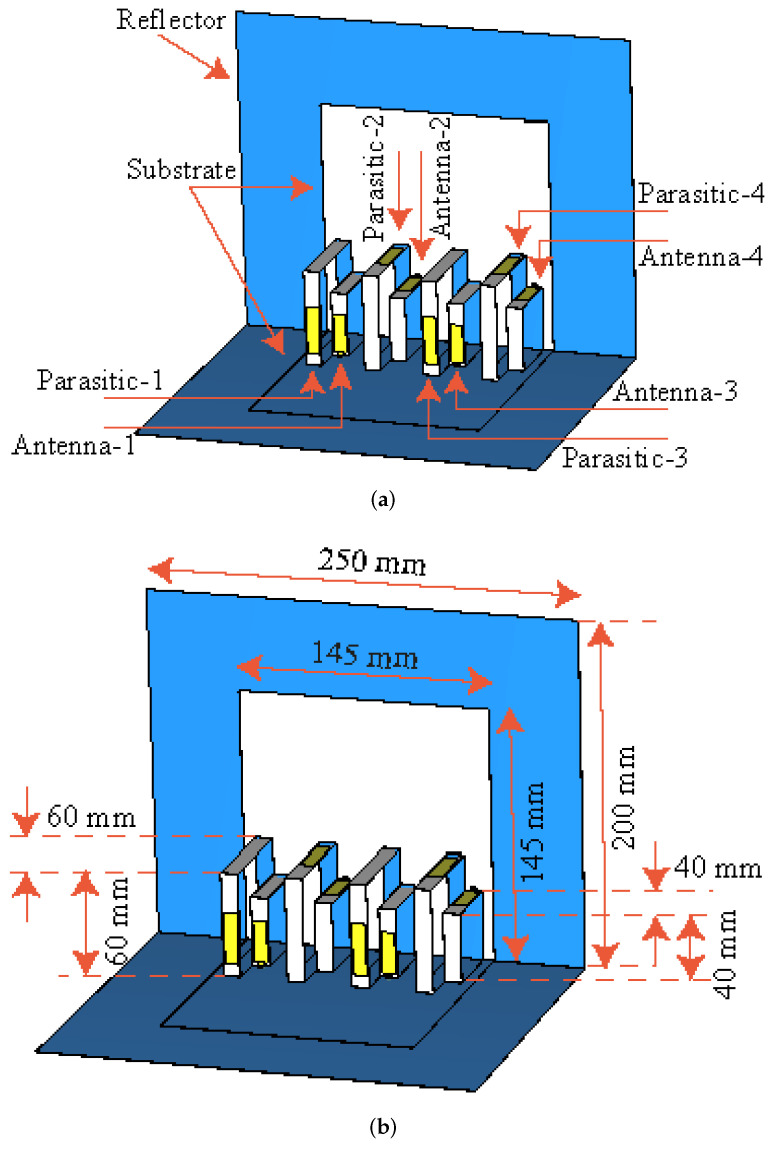
Front view of the proposed Kirigami MIMO antenna (**a**) with each element label and (**b**) with the dimensions.

The design of the proposed Kirigami MIMO antenna is shown in Figure 1. It consists of four rectangular-shaped monopoles and parasitic elements, as shown in Figure 1a. Each radiating and parasitic element is composed of a rectangular-shaped stub. The dimensions of the radiating elements are noted to be $\lambda_{g}$/4, where $\lambda_{g}$ is the guided wavelength and can be calculated using the expression given below.

(1) $$\lambda_{g}=\frac{\lambda_{0}}{\sqrt{\epsilon_{reff}}}$$

where

(2) $$\lambda_{0}=\frac{c}{f_{r}}\;\mathrm{and}\;\epsilon_{reff}=\frac{\epsilon_{r}+1}{2}$$

where $\lambda_{0}$ is the free-space wavelength at the resonant frequency ($f_{r}$) of 2.5 GHz, *c* is the speed of light in free space, and $\epsilon_{r}$ and $\epsilon_{reff}$ are the relative and effective relative permittivity of the dielectric substrate, respectively.

The distance between the radiator and the parasitic element is kept at 20 mm. The purpose of each parasitic element is to tune the resonant frequency and to align the beam in a tilted direction. A 50 Ω coaxial connector is used to feed the radiating elements. For the design of the proposed MIMO antenna, a soft printed board was used, which was called FCCL. The dielectric constant of the FCCL was 3.5 and the dimensions of the sheet were chosen to be 145 mm × 145 mm × 0.25 mm, as shown in Figure 1b. The MIMO antenna system also consisted of a reflector with dimensions of 200 mm × 250 mm (see Figure 1b). The reflector acted as a ground plane and enhanced the gain in the band of interest. For simplicity and clarity, the design was patterned on a paper sheet. Some of the design dimensions are also listed in Table 1.

**Figure 2:**
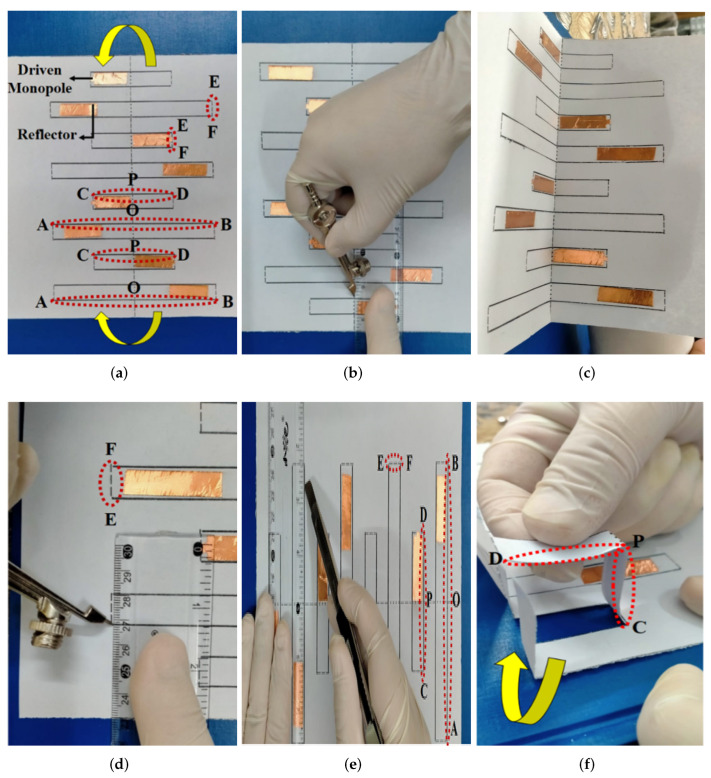
Fabrication steps for the Kirigami MIMO antenna system: (**a**) printed patterns and copper film; (**b**) cut vertical lines; (**c**) folded in half; (**d**) vertical slicing; (**e**) horizontal slicing; (**f**) folded along the horizontal lines to form a staircase geometry.

**Figure 3:**
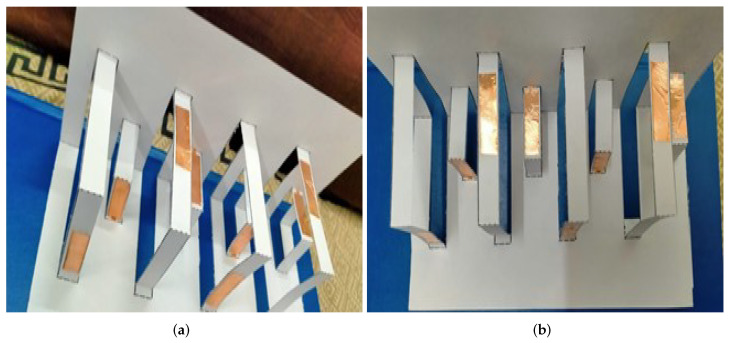
Kirigami MIMO antenna: (**a**) perspective view; (**b**) front view.

**Figure 4:**
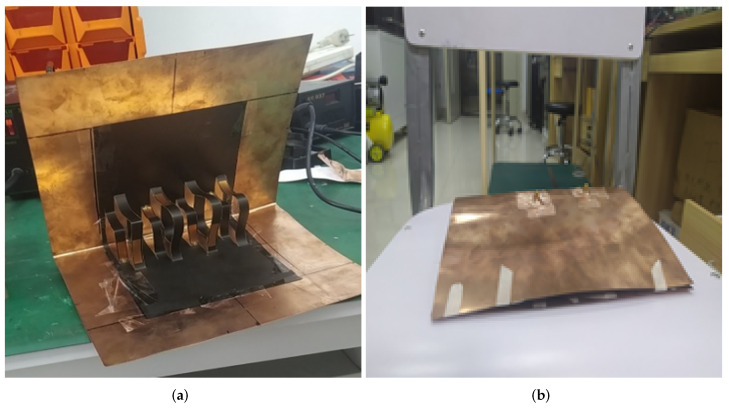
Fabricated prototype of the Kirigami MIMO antenna: (**a**) unfolded state; (**b**) folded state.

For the fabrication of the proposed Kirigami MIMO antenna, the following steps were involved. First, the patterns of radiating and parasitic elements were drawn on a paper sheet, as shown in Figure 2a. After that, they were realized and attached to the sheet using a 0.1-mm-thick copper tape (see Figure 2a). The copper tape used for the design had a low conductivity of 4.4 × 10${}^{5}$ S/m [11]. Next, the sheet was segmented in both directions, i.e., horizontal and vertical. The horizontal segments were named AOB and CPD and are represented by solid lines, while the vertical segment named EF is represented by dashed lines, as shown in Figure 2a. The distance from A to B was 120 mm and that from C to D was 80 mm, whereas O and P were mid-points (see Figure 2a). The first fold was introduced by folding the sheet in the middle of the vertical line, as shown in Figure 2b,c. Next, the vertical segment EF was sliced sharply with a compass (see Figure 2d), followed by the horizontal segments, which were cut with a standard cutter starting from the lower left end of the sheet to the upper right end of the Kirigami pattern, as shown in Figure 2e. Moreover, from the lower left quarter of the pattern, the first column was folded (this could also be done from the upper left quarter of the pattern). Therefore, the horizontal row of CPD formed an inverted L-shaped three-dimensional (3D) pattern (see Figure 2f) or a staircase pattern to ensure swift and smooth operation. In summary, the proposed Kirigami antenna consisted of eight staircases, as shown in Figure 3a. The radiating elements (driven monopoles) were on the smaller staircases, while the parasitic elements were on the larger staircases, 7 mm apart vertically from the monopoles (see Figure 3a,b). Figure 4a shows the fabricated prototype of the proposed Kirigami antenna, while Figure 4b illustrates the fabricated prototype in a folded condition.

## 3. Results and Discussion

**Figure 5:**
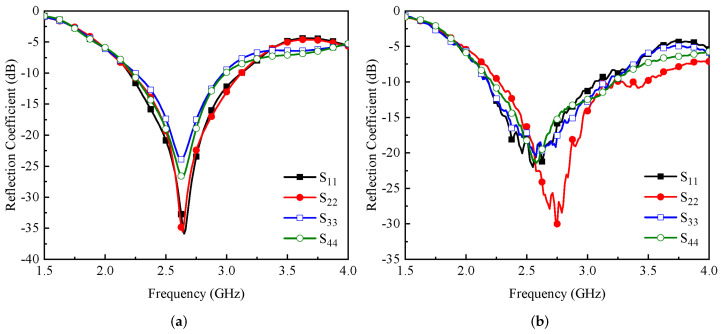
(**a**) Simulated and (**b**) measured reflection coefficients of the MIMO antenna elements.

**Figure 6:**
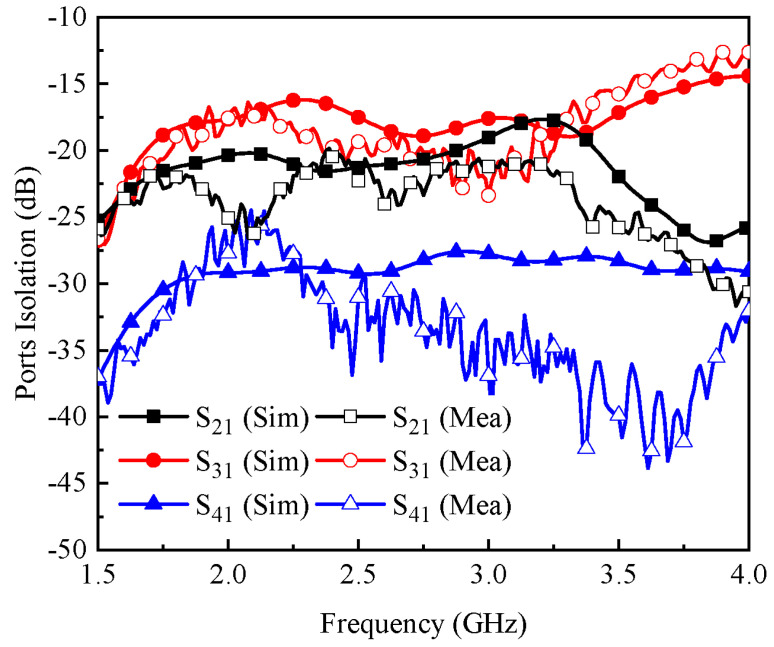
Simulated and measured isolation between the MIMO antenna elements.

The proposed MIMO antenna system was designed and simulated using full-wave electromagnetic software by Computer Software Technology (CST) and measured using an anechoic chamber and a vector network analyzer (VNA). Figure 5a,b illustrate the simulated and measured reflection coefficients from port-1 to port-4. It was observed that the proposed MIMO antenna system had a simulated operating bandwidth of 920 MHz ranging from 2.2 to 3.12 GHz, as shown in Figure 5a, while the measured impedance bandwidth was observed to be 860 MHz in the frequency range of 2.19–3.05 GHz, as shown in Figure 5b. From Figure 5, one can see that the isolation between antenna 1 and antenna 2 was well below 20 dB, and for antenna 1 and antenna 3, it was below 15 dB for the desired frequency band. In addition, the isolation between antenna 1 and antenna 4 was noted to be ≥25 dB. One can also observe from the results of Figure 5 and Figure 6 that excellent agreement was noted between the simulated and measured data. Some discrepancies were observed between the simulated and measured data, which could possibly have arisen due to fabrication tolerances and measurement setup losses.

**Figure 7:**
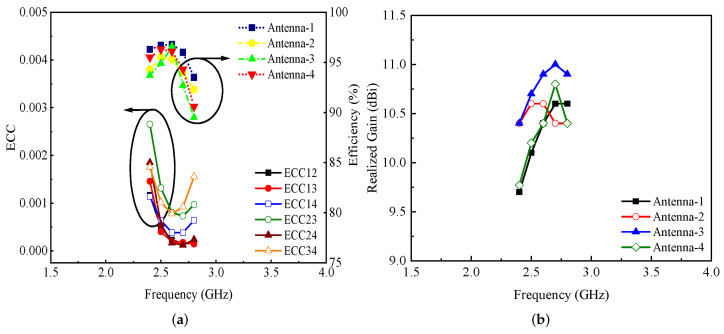
(**a**) ECC, efficiency, and (**b**) realized gain of the proposed Kirigami MIMO antenna.

To demonstrate the potential of the proposed work for modern MIMO-based communication devices, the efficiencies and one of the important key performance parameters, the ECC, were calculated and are shown in Figure 7a. It was observed that the efficiencies of all of the antenna elements were above 90% for the entire operational bandwidth, as shown in Figure 7a. On the other hand, the ECC of the proposed MIMO antenna system was noted to be <0.003 (see Figure 7a). The realized gain of the proposed MIMO antenna is shown in Figure 7b. It can be observed from the figure that the gain of antenna 1 and antenna 4 for the desired frequency band was >9.5 dBi (see Figure 7b), while the gain values of antenna 2 and antenna 3 were around 10.5 and 11 dBi, as shown in Figure 7b.

**Figure 8:**
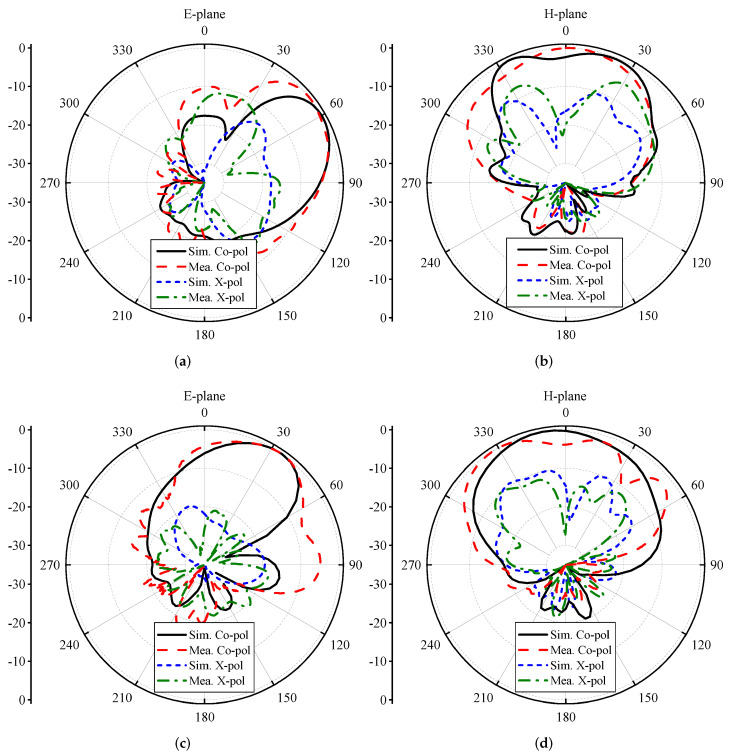
Radiation characteristics of the proposed Kirigami MIMO antenna for (**a**,**b**) antenna 1 and (**c**,**d**) antenna 2.

**Figure 9:**
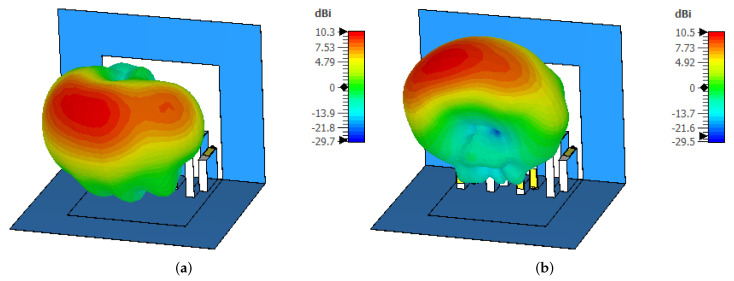
Three-dimensional patterns of the proposed Kirigami MIMO antenna for (**a**) antenna 1 and (**b**) antenna 2.

The far-field radiation characteristics of the proposed MIMO antenna are illustrated in Figure 8. For clarity, the radiation characteristics of antennas 1 and 2 are discussed in both the E- and H-planes. The E-plane co-polarized component of antenna 1 was directional, with a beam tilted towards 60${}^{\circ }$ (see Figure 8a), whereas the magnitude of the cross-polarized (X-pol) component was well below −25 dB, as shown in Figure 8a. As shown in Figure 8b, the co-polarized component of the H-plane had a major lobe directed at θ = 0${}^{\circ }$, while the X-pol component had a null at θ = 0${}^{\circ }$. It was noted that the beamwidth in the H-plane for antenna 1 was around 90${}^{\circ }$. The same kind of pattern was observed for antenna 2 (see Figure 8c,d), but in this case, the E-plane co-polarized component was directed towards 30${}^{\circ }$, as depicted in Figure 8c. The shift in beam position in the E-plane could possibly have been associated with the placement of the antenna elements. From the results, it can also be observed that the proposed MIMO antenna was able to provide polarization diversity characteristics for the band of interest. This effect can be clearly seen from the results of Figure 9, where the 3D radiation characteristics of antenna 1 and antenna 2 are plotted.

**Figure 10:**
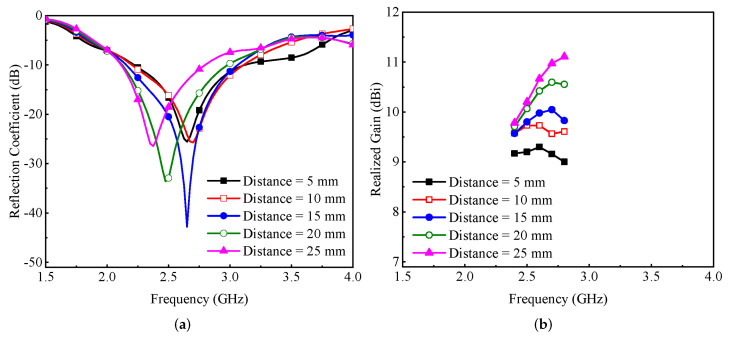
Effect of the distance between the radiating element and parasitic element on the antenna’s (**a**) reflection coefficient and (**b**) gain.

A parametric study was conducted to understand the behavior of the proposed MIMO antenna design. For this purpose, the distance between the radiating element and the parasitic element was changed from 5 to 25 mm, and deviations in the reflection coefficient and gain were observed. As shown in Figure 10a, increasing the distance between the radiating element and the parasitic element caused the resonant frequency to shift towards lower frequency bands, resulting in an increase in wavelength. In addition, with the increase in distance, a reduction in impedance matching was observed, as shown in Figure 10a. However, as shown in Figure 10b, the gain increased as the distance between the radiating element and the parasitic element increased. Here, a trade-off was made between impedance matching and gain, and according to the presented study, a value of 20 mm was selected where the antenna provided a response at 2.5 GHz with acceptable gain.

A comparison of the proposed Kirigami MIMO antenna with previously published work is given in Table 2. As the designed MIMO antenna was large in size, it provided higher gain than that of the designs presented in [10,11,15,16,17]. In addition, the design offered a higher or equal value of isolation compared to the designs of [10,15,17].

## 4. Conclusions

This work reports a low-cost and deployable Kirigami-based MIMO antenna system for sub-6 GHz applications. The designed MIMO antenna system consists of four radiating and four parasitic elements backed by a folded reflector. The proposed MIMO antenna was fabricated using a low-cost FCCL sheet and copper tape. Detailed analyses of different aspects, fabrication steps, and key performance parameters of the antenna system were discussed and presented. The designed MIMO antenna system operates well in the 2.5 GHz frequency band and provides an impedance bandwidth of 860 MHz. It exhibits a high gain of ≈11 dBi with an efficiency of more than 90% and an ECC well below 0.003.

## Figures and Tables

**Table 1:** Design dimensions of the proposed Kirigami MIMO antenna.

Parameter	Value (mm)	Parameter	Value (mm)
AOB	120	Radiating element length	24
CPD	80	Radiating element width	8
EF	10	Reflector Length	200
Parasitic element length	30	Reflector width	250
Parasitic element width	8	−	−

**Table 2:** Comparison between the proposed and previously presented Kirigami and Origami antennas.

Ref.	Antenna Elements	Size (mm${}^{2}$)	Frequency Band (GHz)	Bandwidth (GHz)	Isolation (dB)	Gain (dBi)	Substrate	Complexity	Folding State
[10]	2	60 × 60	2.5/3.2	0.2/0.25	10	6.15	Ecoflex	Simple	Available
[11]	1	120 × 120	0.15	0.02	−	1.53	FR-4/Paper	High	Available
[15]	2	210 × 210	2.4/5.2	0.45/1.6	15	2.78	Paper	High	Available
[16]	2	50 × 70	2.4/2.91/ 3.27/3.4/5.1	0.48/1.08/ 0.7/0.4/0.7	30	4	FR-4	High	Not Available
[17]	4	38 × 90	3.6	0.62	10	4.5	RO4350B	Simple	Not Available
Proposed	4	250 × 200	2.5	0.86	15	11	FCCL/Paper	Simple	Available
